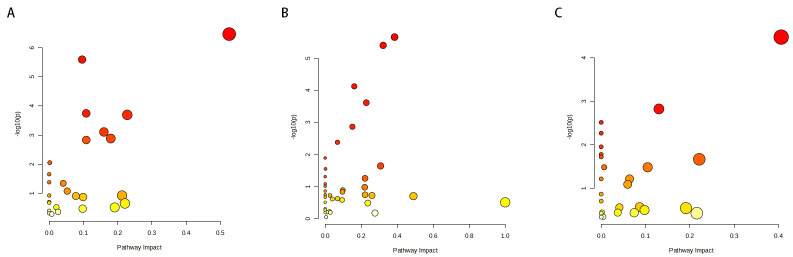# Causal inference of metabolites in autoimmune liver diseases: A Mendelian randomization analysis

**DOI:** 10.1097/MD.0000000000048221

**Published:** 2026-05-08

**Authors:** Xing Ren, Wenna Yang, Xiuli Yan, Hui Zhang

**Affiliations:** a Institute of Interdisciplinary Integrative Medicine Research, Shanghai University of Traditional Chinese Medicine, Shanghai, China; b Yueyang Hospital of Integrated Traditional Chinese and Western Medicine, Shanghai University of Traditional Chinese Medicine, Shanghai, China.

**Keywords:** autoimmune hepatitis, autoimmune liver disease, Mendelian randomization, metabolites, primary biliary cirrhosis, primary sclerosing cholangitis

## Abstract

Autoimmune liver disease (AILD) is a chronic and insidious liver disease that can lead to cirrhosis. Metabolites have been shown to play an essential role in autoimmune diseases, but the causality between metabolites and AILD has not been completely investigated. This Mendelian randomization (MR) analysis was conducted to evaluate the causal relationship of metabolites on AILD. A genome-wide association study was conducted using 1400 metabolites as the exposure, and 3 AILD phenotypes primarily from European sources were identified as the results. Inverse-variance weighted method was implemented to obtain the primary causal estimates. Inverse-variance weighted, MR-Egger, weighted median and MR-PRESSO methods were employed for sensitivity analysis. In addition, metabolic pathway analysis was conducted with the utilization of MetaboAnalyst 6.0. All of the statistical analysis was carried out using the R software. In our 2-sample MR analysis, 262 metabolites that may be causally related to the pathogenesis of AILD were identifie. Metabolites strongly associated with the development of AILD, such as plasma free proline, spermidine to N-acetylputrescine ratio and hexadecanedioate (C16-DC) were identified by sensitivity analysis. Metabolic pathway analysis detected 31 prominent metabolic pathways in AILD. Our study provides new evidence for the association between 1400 metabolites and 3 types of AILD. It is worthy of exploration whether metabolites with causality can serve as biomarkers to distinguish patients at high risk of AILD.

## 1. Introduction

Autoimmune liver disease (AILD) is a chronic liver disease caused by autoimmune disorders of unknown etiology, characterized by a loss of immunological tolerance to hepatocytes and biliary epithelial cells.^[1,2]^ It includes 3 main clinical entities: autoimmune hepatitis (AIH), primary biliary cirrhosis (PBC) and primary sclerosing cholangitis (PSC).^[3]^ Although the incidence of AILD is low, reported to be <50 per 100,000,^[4]^ it is a common cause of chronic hepatitis, leading to cirrhosis due to occult onset.^[5]^ Treatment usually involves the use of immunosuppressive drugs as well as some newly developed biologics, which are effective but also prone to therapeutic resistance and can cause adverse side effects with long-term use.^[6]^

Metabolites, which are the final resultant products of gene and protein expression, are capable of bringing about persistent alterations in cellular organization and genetic structure via epigenomic modulation.^[7]^ The link between metabolism and epigenetics is supported by growing evidence that metabolic pathways and products help determine minute-to-minute cellular phenotypes, influencing epigenetics and gene transcription.^[8,9]^ It was found that abnormal energy metabolism may disrupt immune tolerance and lead to an autoimmune response.^[10]^ In addition, immune cells exhibit a high degree of sensitivity to metabolites in the body, such as being able to quickly adapt to the microenvironment of low-glycemic and high-lactate, thereby controlling metabolic homeostasis and maintaining immune tolerance to themselves.^[11,12]^ Previous investigations have suggested the essential role of metabolites in AILD. For example, serine may reduce the risk of AIH, while glycine may increase the risk of PBC.^[13,14]^ However, traditional observational studies are likely to be influenced by external conditions, rendering it arduous to ascertain the causality between metabolites and AILD. Besides, as the continuous updating and increase of metabolites in the database, new findings are expected to emerge.

Mendelian randomization (MR) analysis is an analytic methodology in which genetic variants are used as instrumental variables (IVs) to assess genetic causality between risk factors and outcomes.^[15]^ Because genetic variants are randomly assigned during gamete formation and conception, MR analysis minimizes the influence of reverse causality.^[16]^ Notably, large-scale Genome-wide association study (GWAS) analysis make it feasible to search metabolites that are causally related to AILD from a genetic perspective.^[17]^ In this research, 2-sample MR analysis was performed to assess the causality between 1400 metabolites and 3 types of AILD, including AIH, PBC, and PSC. And based on the metabolites, to further identify metabolic pathways that may play essential roles in the development of AILD.

## 2. Materials and methods

### 2.1. Study design

**Figure 1.**
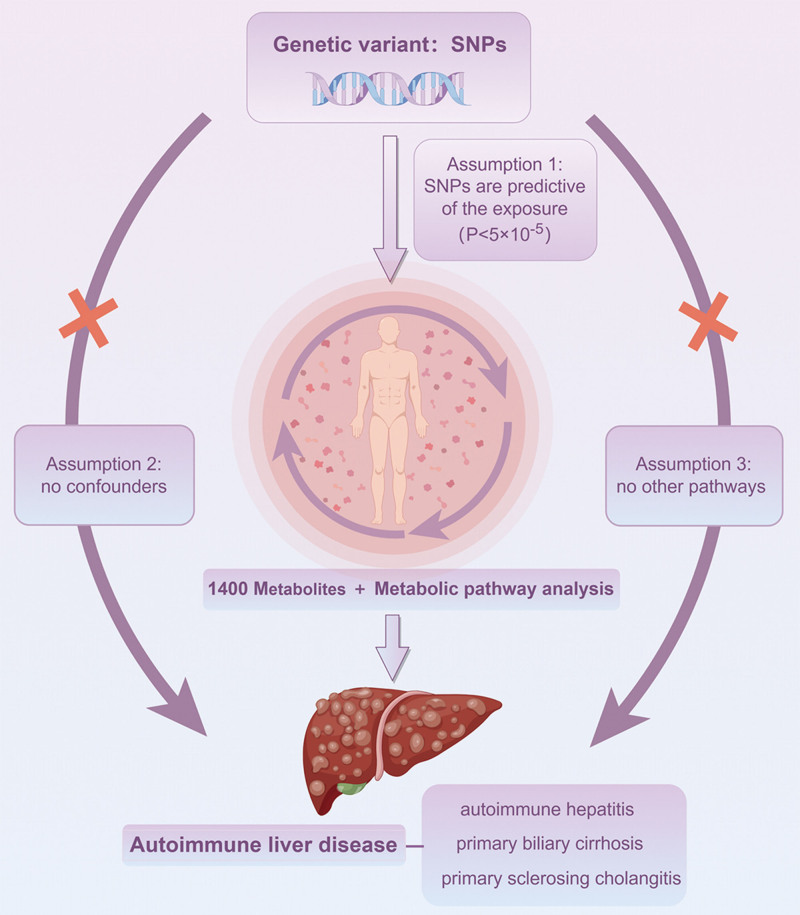
An overall design of the present study. Created with FigDraw (www.figdraw.com).

This MR analysis was conducted to evaluate the causal relationship of 1400 metabolites on AILD. To obtain more credible results using the MR analysis, our study aims to meet 3 assumptions. First of all, the IVs should have a notable correlation with metabolites. Secondly, the IVs should not be related to other confounding elements. Finally, apart from the exposure factors, the IVs ought not to influence the outcome via alternative pathways.^[18]^ A workflow of our study is illustrated in Figure 1.

### 2.2. GWAS data sources for AILD and metabolites

We used GWAS pooled data for this study (http://gwas.mrcieu.ac.uk). In our analysis, we used publicly available sets of summary statistics obtained from GWAS performed on individuals diagnosed with AIH, PBC, and PSC, as detailed in Table 1. Various metabolite GWAS statistics have been deposited in the GWAS catalogue (https://www.ebi.ac.uk/gwas/), which cover 1400 metabolites.

**Table 1:** Characteristics of the summary datasets for AILD.

AILD types	Population	Sample size	Case	Control	SNPs	PMID
AIH	European	485,234	821	484,413	24,198,482	34594039
PBC	European	24,510	8021	16,489	5004,018	34033851
PSC	Mixed	14,890	2871	12,019	7891,603	27992413

### 2.3. IVs selection

We set a significance level of 5 × 10^−5^ for the independent variables each metabolite, which aligned with previous study.^[19]^ To avoid linkage imbalance, we clustered these SNPs (kb = 500, *r*^2^ = 0.01). We computed the proportion of variance exposed by employing the *R*^2^ value of each SNP and utilized the *F*-statistic to estimate instrument strength and avoid weak instrument bias.

### 2.4. MR analysis

The causality between metabolites and AILD was mainly evaluated by inverse variance weighted (IVW) method. The IVW method, which is a fundamental approach in MR studies, aggregates all the Wald ratios corresponding to each SNP in order to obtain summary estimates. The IVW method has significantly higher statistical power than other MR methods but is also prone to pleiotropy bias. Notably, causality is reliable if 3 additional MR tests are performed. Therefore, sensitivity analysis was performed: the weighted median-based method to assess the robustness of the significant associations found; the MR-Egger regression to estimate horizontal pleiotropy, by which genetic variation was independently correlated with exposure and outcome; and the MR-PRESSO test was utilized to evaluate horizontal pleiotropy and to scrutinize potential instrumental outliers.

### 2.5. Metabolic pathway analysis

After MR analysis, we next used the Human Metabolome Database (HMDB, http://www.hmdb.ca/) to search the HMDB IDs of metabolites which were identified by IVW at *P* < .05. Pathway enrichment analysis was performed using MetaboAnalyst 6.0 (https://www.metaboanalyst.ca/) based on HMDB IDs.

### 2.6. Statistical analysis

Statistical analyses were performed in R (version 4.2.2), and MR analysis were conducted via the TwoSampleMR package. We conducted MR-PRESSO test by using the MR-PRESSO package. Besides, false discovery rate (FDR) corrections were performed to address the issue of false positives that arose from multiple testing.

## 3. Results

### 3.1. Study overview

We implemented a 2-sample MR analysis, employing GWAS summary statistics, to evaluate the causal effects of metabolites on 3 types of AILD. To evaluate the causality between each metabolite and the outcomes, we selected genetic variation as IVs. The study design is presented in Figure 1. Importantly, the minimum F statistic for validity tests of these genetic predictors was 19.88, indicating that all IVs for the 1400 kinds of metabolites were sufficiently credible.

### 3.2. Causal effect of the 1400 metabolites on AILD

**Figure 2.**
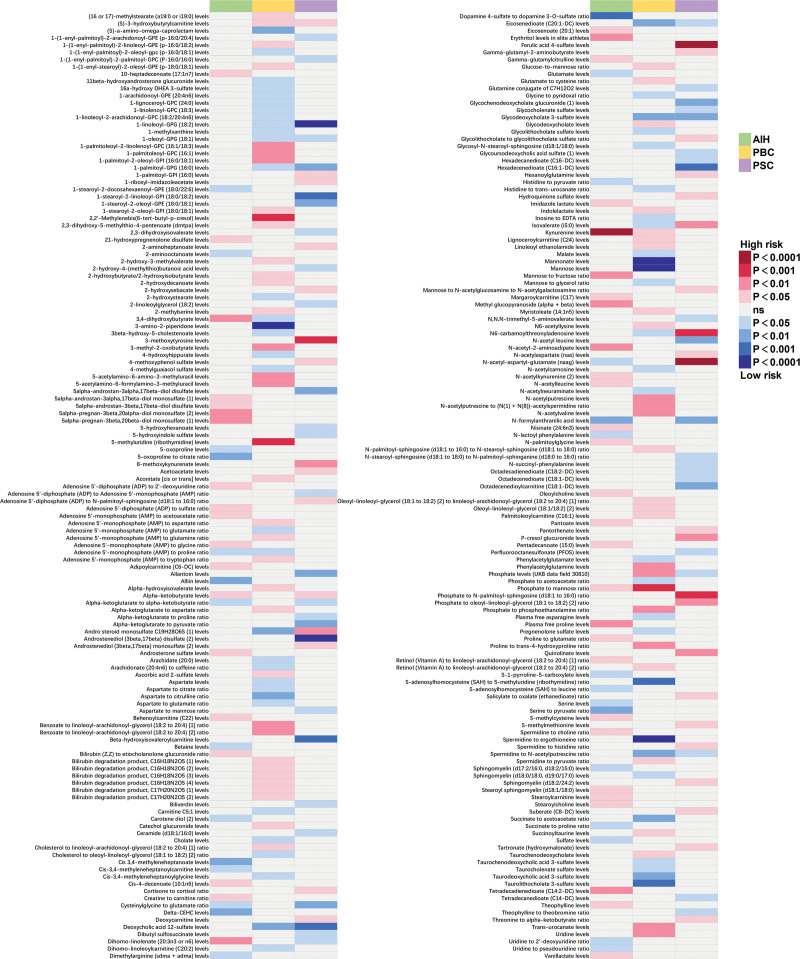
Mendelian randomization associations of known metabolites on the risk of the 3 phenotypes of AILD. (derived from the fixed-effect IVW analysis).

The IVW approach was utilized to explore the causality between AILD and 1400 metabolites. A total of 262 significant pathogenic association features were identified at *P* < .05: 76 for AIH, 123 for PBC, and 85 for PSC, and all significant associations between metabolites and AD are shown in Figure 2. We observed that some AILD types share common causal metabolites (Supplementary Figure 1 and Supplementary Table 1). Alpha-ketobutyrate was was linked to a heightened risk in AIH and PSC (odds ratio [OR]: 1.35, 95% confidence interval [CI]: 1.01–1.81, *P* = .042; OR: 1.35, 95% CI: 1.02–1.79, *P* = .039), while N-formylanthranilic acid was associated with a decreased risk (OR: 0.73, 95% CI: 0.58–0.91, *P* = .004; OR: 0.77, 95% CI: 0.63–0.94, *P* = .010). Deoxycholic acid 12-sulfate showed inverse associations with the risk of PBC and PSC (OR: 0.85, 95% CI: 0.76–0.96, *P* = .007; OR: 0.80, 95% CI: 0.70–0.90, *P* = 4E-04). Kynurenine had a suggestive negative effect on AIH and PBC (OR: 1.73, 95% CI: 1.39–2.15, *P* = 1.03E-06; OR: 1.25, 95% CI: 1.01–1.54, *P* = .038). Interestingly, there may be different risk implications between the same metabolite and AILD. Isovalerate was correlated with a reduced risk in PBC (OR: 0.79, 95% CI: 0.64–0.98, *P* = .031), whereas it was correlated with an increased risk of PSC (OR: 1.52, 95% CI: 1.12–2.08, *P* = .008). Phosphate was associated with an increased risk in PBC (OR: 1.29, 95% CI: 1.06–1.56, *P* = .010), whereas it was associated with a decreased risk of PSC (OR: 0.81, 95% CI: 0.67–0.99, *P* = .040). Dihomo-linolenate showed an inverse correlation with the risk of PSC (OR: 0.73, 95% CI: 0.58–0.93, *P* = .011) and it showed positive association with AIH risk (OR: 1.40, 95% CI: 1.10–1.79, *P* = .007). Besides, 3,4-dihydroxybutyrate had a suggestive positive effect on the risk of PBC (OR: 0.74, 95% CI: 0.57–0.95, *P* = .020) but a negative effect on AIH (OR: 1.52, 95% CI: 1.11–2.09, *P* = .009) cues.

### 3.3. Results of sensitivity analysis

**Figure 3.**
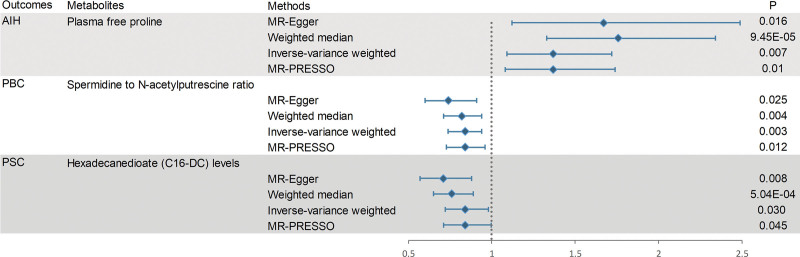
Sensitivity analysis for significant metabolites on AILD. (A) Sensitivity analysis between AIH and plasma free proline. (B) Sensitivity analysis between PBC and spermidine to N-acetylputrescine ratio. (C) Sensitivity analysis between PSC and Hexadecanedioate (C16-DC).

**Figure 4.**
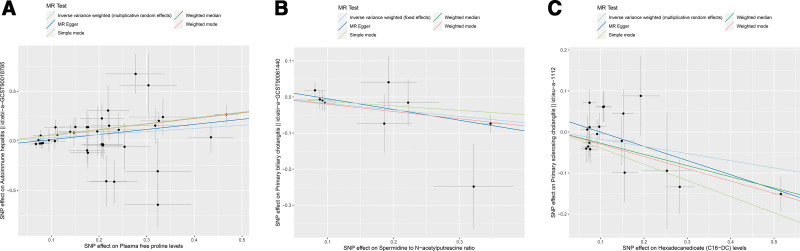
Scatter plots for the 3 metabolites on the risk of 3 AILD phenotypes. (A) Plasma free proline on AIH. (B) Spermidine to N-acetylputrescine ratio on PBC. (C) Hexadecanedioate (C16-DC) on PSC.

**Figure 5.**
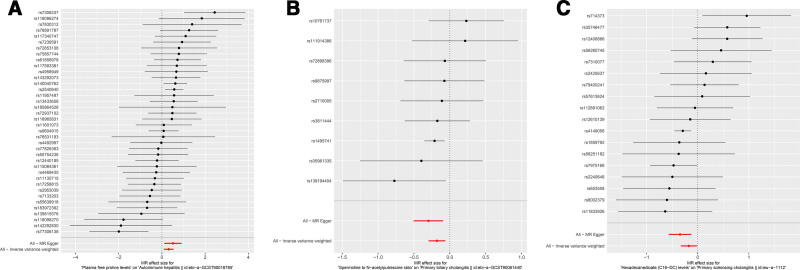
Forest plots for the 3 metabolites on the risk of 3 AILD phenotypes. (A) Plasma free proline on AIH. (B) Spermidine to N-acetylputrescine ratio on PBC. (C) Hexadecanedioate (C16-DC) on PSC.

Although the IVW method is highly proficient in deducing the causal relationship between exposures and outcomes, it is susceptible to instrumental bias. Thus, sensitivity analysis is used to further evaluate the robustness of causal relationships. Figure 3 shows the results of the sensitivity analysis for 3 pairs of metabolites and AILD with significant causal relationships. Causality is reliable if 3 additional MR tests are performed. After the validation of 4 approaches (IVW, MR-Egger, weighted median and MR-PRESSO), Plasma free proline is a high risk factor for AIH, Spermidine to N-acetylputrescine ratio is a low-risk factor for PBC and Hexadecanedioate (C16-DC) is a low-risk factor for PSC (all the *P *< .05). Scatter plots and forest plots are shown in Figures 4 and 5. Funnel plots and leave-one-out analysis plot are shown in Supplementary Figures 2 and 3. The complete results of the pleiotropy and heterogeneity analysis are listed in Supplementary Tables 2 and 3.

### 3.4. Metabolic pathway analysis

**Figure 6.**
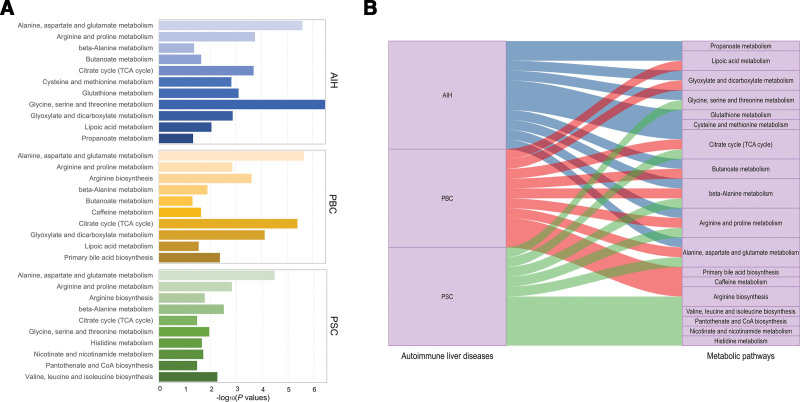
Significant metabolic pathways of 3 AILD phenotypes. (A) Bar plot of metabolite pathways enrichment. (B) Sankey plot of metabolite pathways distribution.

Metabolic pathway analysis revealed 31 prominent metabolic pathways within AILD (Fig. 6A). Among them, 11 pathways are enriched in AIH, 11 pathways are enriched in PBC, and 10 pathways are enriched in PSC. The details are shown in Table 2 and Supplementary Figure 4. It is worth noting that there are some common key metabolic pathways in 2 or more types of AILD (Fig. 6B).

**Table 2:** Significant metabolic pathways involved in different AILD phenotypes.

Metabolite set	Total	Hits	Expected	*P* value	FDR
AIH					
Glycine, serine, and threonine metabolism	33	7	0.524	3.35E-07	2.68E-05
Alanine, aspartate, and glutamate metabolism	28	6	0.444	2.52E-06	0.0001
Arginine and proline metabolism	36	5	0.571	.0002	0.004
Citrate cycle (TCA cycle)	20	4	0.317	.0002	0.004
Glutathione metabolism	28	4	0.444	.001	0.013
Glyoxylate and dicarboxylate metabolism	32	4	0.508	.001	0.017
Cysteine and methionine metabolism	33	4	0.524	.001	0.017
Lipoic acid metabolism	28	3	0.444	.009	0.089
Butanoate metabolism	15	2	0.238	.022	0.199
Beta-alanine metabolism	21	2	0.333	.042	0.335
Propanoate metabolism	22	2	0.349	.046	0.335
PBC					
Alanine, aspartate, and glutamate metabolism	28	7	0.676	2.20E-06	0.0002
Citrate cycle (TCA cycle)	20	6	0.483	3.98E-06	0.0002
Glyoxylate and dicarboxylate metabolism	32	6	0.772	.0001	0.002
Arginine biosynthesis	14	4	0.338	.0002	0.005
Arginine and proline metabolism	36	5	0.869	.001	0.022
Primary bile acid biosynthesis	46	5	1.110	.004	0.055
Beta-alanine metabolism	21	3	0.507	.013	0.146
Caffeine metabolism	10	2	0.241	.023	0.226
Lipoic acid metabolism	28	3	0.676	.028	0.249
Butanoate metabolism	15	2	0.362	.049	0.391
PSC					
Alanine, aspartate, and glutamate metabolism	28	5	0.409	3.30E-05	0.003
Arginine and proline metabolism	36	4	0.526	.001	0.060
Beta-alanine metabolism	21	3	0.307	.003	0.081
Valine, leucine, and isoleucine biosynthesis	8	2	0.117	.005	0.108
Glycine, serine, and threonine metabolism	33	3	0.482	.011	0.179
Arginine biosynthesis	14	2	0.204	.017	0.216
Nicotinate and nicotinamide metabolism	15	2	0.219	.019	0.216
Histidine metabolism	16	2	0.234	.022	0.216
Pantothenate and CoA biosynthesis	20	2	0.292	.033	0.264
Citrate cycle (TCA cycle)	20	2	0.292	.033	0.264

## 4. Discussion

AILD is a chronic liver condition characterized by an autoimmune response with an unknown etiology.^[1]^ Although its pathological mechanisms remain unclear, mounting evidence highlights the connection between blood metabolites and the risk of autoimmune disorders. In this study, we conducted a MR study to analyze the causal relationships between 1400 metabolites. Key findings include both shared and distinct metabolic influences across AILD subtypes. Sensitivity analyses confirmed robust causal roles for specific metabolites, including plasma free proline as a risk factor for AIH and spermidine to N-acetylputrescine ratio as protective for PBC. Additionally, metabolic pathway analysis revealed 31 significantly enriched pathways, with several common across multiple AILD subtypes, highlighting shared metabolic mechanisms in disease pathogenesis. As far as we know, this is the first MR study to systematically assess the causal role of metabolites in AILD. The following text will discuss the research results.

Our study showed a group of metabolites related to AIH, among which plasma free proline had a strong effect on AIH. Proline, whose unique function contributes to the special role proline plays in protein structure and function.^[20]^ Studies have reported that mutations in the proline molecule may lead to autoimmune diseases such as autoimmune hemolytic anemia^[21]^ and AIH.^[22]^ Therefore, maintaining the stability of proline metabolism may be important for AIH. PBC and PSC are one of the AILD, presenting as a chronic inflammation of the bile ducts, with an increase in morbidity and mortality.^[23]^ Spermidine is a naturally occurring polyamine that is essential for cell survival, proliferation, differentiation, and senescence.^[24]^ It is noteworthy that spermidine has been found to be associated with autoimmune diseases.^[25]^ Hexadecanedioate is a byproduct of omega oxidation of fatty acids, which is a secondary pathway for fatty acid oxidation when beta oxidation is insufficient.^[26]^ In previous studies, hexadecanedioate was often associated with blood pressure regulation, and high circulating levels of hexadecanedioate may lead to hypertension.^[26]^ Notably, new research has found that hexadecanedioate is thought to be a protective factor in some autoimmune diseases, such as rheumatoid arthritis.^[14]^ The effects of hexadecanedioate needs to be explored further. In the present study, hexadecanedioate has a causal relationship with PSC and may be a potential low-risk factor.

Our study also uncovered metabolic pathways that may be associated with AILD. The results suggest that there may be a close link between multiple amino acid metabolic pathways and AILD. The amino acid is the constituent unit of proteins and is important for human physiological functions.^[27]^ Abnormalities in amino acid metabolic pathways have been associated with the development of a variety of diseases, such as acute liver injury and tumors.^[28]^ Arginine is a semi-essential amino acid that participates in the urea cycle as well as arginine/proline metabolism. In addition to being the cornerstone of proteins, arginine has been selected as a metabolic key point in the immune system to regulate immune responses.^[29]^ Arginine metabolism has recently become a key pathway in the control of immune cell function, such as activation of T cells.^[30]^ Arginine can produce nitric oxide (NO) and citrulline via the nitric oxide synthase pathway.^[31]^ Available studies showed that NO and its redox derivatives may attenuate immune cell infiltration by inhibiting multiple inflammatory pathways,^[32]^ which may be protective against autoimmune diseases. Glycine is believed to significantly inhibit the activation of inflammatory cells, such as directly activating the glycine gated chloride (Cl^-^) channels (GlyR) that are presented in inflammatory cells, like macrophages^[33]^ and reducing the production of proinflammatory cytokines.^[34]^ Some studies have found that the concentration of glycine in urine increases during the active phase of autoimmune diseases and decreases during the remission phase.^[35]^ In addition, studies have shown that glycine increases the risk of AILD.^[14]^ We speculate that glycine may serve as a risk factor and indirectly reflect a certain inflammatory state. It is worth noting that elevated levels of aspartate and alanine aminotransferase are a feature of AIH^[36]^ and a high ratio of aspartate to alanine aminotransferase serves as an indication cirrhosis and poor prognosis in PSC.^[37]^ This could be potential proof that aspartate and alanine are associated with AILD.

The citrate cycle (TCA cycle) is a major metabolic pathway in the mitochondrial matrix, which provides energy to cells by producing ATP with extremely high efficiency. In addition to being involved in metabolism, the TCA cycle is a nexus for a variety of nutrient inputs.^[38]^ Recently, the TCA cycle has been found to be highly relevant to immune metabolism and plays a crucial role in the regulation of immune cell responses.^[38]^ Metabolites of the TCA cycle have a significant impact on immunotherapy, such as citrate. T helper 17 (Th17) cells have been shown to play a key role in the pathogenesis of AIH,^[39]^ PBC,^[40]^ and PSC.^[41]^ Citrate can restore the metabolism and function of pyruvate dehydrogenase-deficient Th17 cells,^[42]^ offering the possibility of targeting Th17 cell-driven AILD. Accumulating evidences have indicated that caffeine is not only a psychoactive drug but also an important immunomodulator.^[43]^ It is well known that B cells and T cells are essential components in autoimmune diseases, and when both cell lines are activated without proper regulation, they can lead to a massive release of cytokines and cause damage to their own tissues.^[44]^ Caffeine consumption has been demonstrated to inhibit the multiplication of Th1 and Th2 cells.^[45]^ Besides, caffeine intake leads to altered B cell function and inhibits antibody production.^[46]^ Therefore, it is reasonable to speculate that the possible immunosuppressive effects of caffeine may be helpful in the condition of AILD.

Our study has several strengths. First, we used the latest updated data on 1400 metabolites to analyze the causality between metabolites and AILD, and the data on AILD came from a total of 11,713 cases, so the sample size of this study was large. Besides, we used a variety of statistical tests with a high degree of confidence. However, our study still has some limitations. Since the functions and mechanisms of certain metabolites in disease remain incompletely comprehended, this restricts our understanding and explanation of the outcomes of MR analysis. In addition, this study was conducted mainly on European populations, so additional validation in various populations is needed in the future.

## 5. Conclusion

In summary, this is the first systematic MR analysis using genome-wide data to assess causal associations between 1400 serum metabolites and 3 AILD phenotypes, offering initial proof regarding the influence of circulating metabolic disorders on the risk of AILD. A total of 262 metabolites that may be causally related to the pathogenesis of AILD were identified by IVW analysis, of which 22 metabolites were causally related to more than one AILD. There are 31 significant metabolic pathways for AILD have been identified through metabolic pathway analysis, 4 of which were strongly associated with all AILDs. Overall, our findings will provide valuable insights into the use of metabolites as potential biomarkers for exploring targeted drugs for the treatment of AILD, but further studies are needed to reveal their role in the pathogenesis of related diseases.

## Acknowledgments

We thank all the GWASs for making the summary data publicly available. And we thank the picture materials by Figdraw (www.figdraw.com).

## Author contributions

**Conceptualization:** Xing Ren, Xiuli Yan, Hui Zhang.

**Data curation:** Wenna Yang, Hui Zhang.

**Formal analysis:** Xiuli Yan.

**Funding acquisition:** Hui Zhang.

**Investigation:** Wenna Yang.

**Methodology:** Xing Ren, Wenna Yang, Xiuli Yan, Hui Zhang.

**Software:** Xing Ren.

**Visualization:** Xing Ren.

**Writing – original draft:** Xing Ren.

**Writing – review & editing:** Wenna Yang, Xiuli Yan, Hui Zhang.

**Figure:**
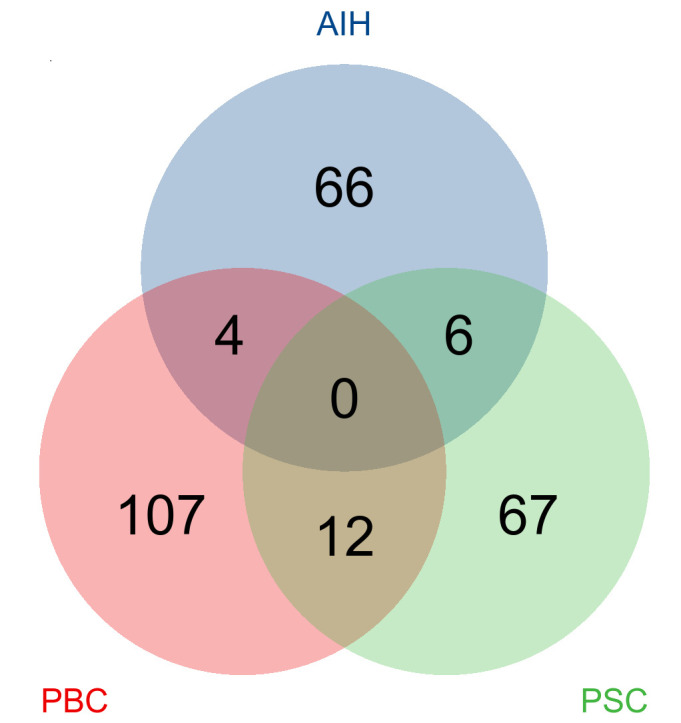




**Figure:**
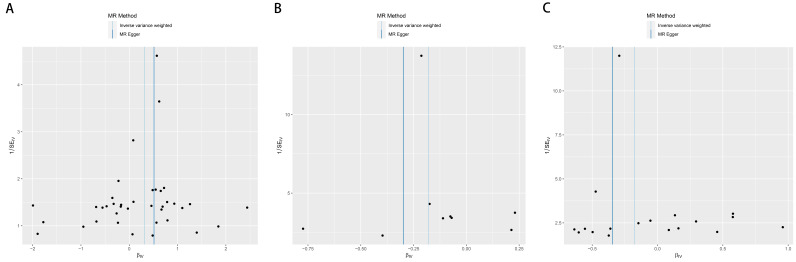




**Figure:**