# Supplementary material for: Causal inference of metabolites in autoimmune liver diseases: A Mendelian randomization analysis
Source: Medicine (Baltimore). 2026 May 8;105(19):e48221. doi: 10.1097/MD.0000000000048221 (PMC13166632; doi:10.1097/MD.0000000000048221)
Supplement: Supplementary file 1 [file medi-105-e48221-s002.docx]

**Table S1** The distribution of significant metabolites in AILD.

| Only AIH | Only PBC | Only PSC | AIH AND PBC NOT PSC | AIH AND PSC NOT PBC | PBC AND  PSC NOT  AIH |
| --- | --- | --- | --- | --- | --- |
| Dopamine 4-sulfate to dopamine 3-O-sulfate ratio | Malate levels | 1-stearoyl-2-linoleoyl-GPI (18:0/18:2) levels | Adenosine 5'-monophosphate (AMP) to proline ratio | N-formylanthranilic acid levels | N6-carbamoylthreonyladenosine levels |
| N-lactoyl phenylalanine levels | (S)-a-amino-omega-caprolactam levels | Androstenediol (3beta,17beta) disulfate (2) levels | Phosphate to mannose ratio | Cysteinylglycine to glutamate ratio | Isovalerate (i5:0) levels |
| Serine to pyruvate ratio | Mannose to glycerol ratio | N-acetyl leucine levels | 3,4-dihydroxybutyrate levels | Alpha-ketoglutarate to alpha-ketobutyrate ratio | Andro steroid monosulfate C19H28O6S (1) levels |
| Delta-CEHC levels | 3-amino-2-piperidone levels | N-succinyl-phenylalanine levels | Kynurenine levels | N-acetyl-aspartyl-glutamate (naag) levels | Spermidine to N-acetylputrescine ratio |
| Succinate to proline ratio | Succinate to acetoacetate ratio | Alpha-ketoglutarate to pyruvate ratio |  | Alpha-ketobutyrate levels | Deoxycholic acid 12-sulfate levels |
| Cis 3,4-methyleneheptanoate levels | Taurolithocholate 3-sulfate levels | Theophylline to theobromine ratio |  | Dihomo-linolenate (20:3n3 or n6) levels | Eicosenedioate (C20:1-DC) levels |
| S-adenosylhomocysteine (SAH) to leucine ratio | 4-hydroxyhippurate levels | Allantoin levels |  |  | 1-palmitoyl-GPG (16:0) levels |
| Glutamate levels | Mannose levels | 2,3-dihydroxyisovalerate levels |  |  | 1-oleoyl-GPG (18:1) levels |
| Uridine to 2'-deoxyuridine ratio | Glycine to pyridoxal ratio | Alpha-ketoglutarate to proline ratio |  |  | 1-linoleoyl-GPG (18:2) levels |
| Sphingomyelin (d17:2/16:0, d18:2/15:0) levels | 1-linolenoyl-GPC (18:3) levels | Glutamine conjugate of C7H12O2 levels | |  | Glycodeoxycholate 3-sulfate levels |
| Alliin levels | 4-methylguaiacol sulfate levels | 5-hydroxyindole sulfate levels |  |  | (S)-3-hydroxybutyrylcarnitine levels |
| S-1-pyrroline-5-carboxylate levels | Mannonate levels | 5-hydroxyhexanoate levels |  |  | Phosphate levels (UKB data field 30810) |
| Uridine to pseudouridine ratio | Phosphate to acetoacetate ratio | 5alpha-androstan-3alpha,17beta-diol disulfate levels | |  |  |
| Carotene diol (2) levels | 1-lignoceroyl-GPC (24:0) levels | Dibutyl sulfosuccinate levels |  |  |  |
| Dimethylarginine (sdma + adma) levels | Cholate levels | 1-stearoyl-2-oleoyl-GPE (18:0/18:1) levels | |  |  |
| 5-oxoproline to citrate ratio | Aspartate to citrulline ratio | 1-(1-enyl-palmitoyl)-2-arachidonoyl-GPE (p-16:0/20:4) levels | |  |  |
| Sulfate levels | S-adenosylhomocysteine (SAH) to 5-methyluridine (ribothymidine) ratio | Beta-hydroxyisovaleroylcarnitine levels | |  |  |
| Histidine to pyruvate ratio | Aspartate to citrate ratio | Tetradecanedioate (C14-DC) levels |  |  |  |
| Betaine levels | N-acetylneuraminate levels | Ceramide (d18:1/16:0) levels |  |  |  |
| Cis-3,4-methyleneheptanoylcarnitine levels | Arachidate (20:0) levels | Perfluorooctanesulfonate (PFOS) levels | |  |  |
| Serine levels | Aspartate to glutamate ratio | N-stearoyl-sphingosine (d18:1 to 18:0) to N-palmitoyl-sphinganine (d18:0 to 16:0) ratio | | |  |
| 5-oxoproline levels | 2-hydroxystearate levels | 2-linoleoylglycerol (18:2) levels |  |  |  |
| 1-stearoyl-2-docosahexaenoyl-GPE (18:0/22:6) levels | Adenosine 5'-monophosphate (AMP) to glutamate ratio | Adenosine 5'-diphosphate (ADP) to Adenosine 5'-monophosphate (AMP) ratio | |  |  |
| 2-aminooctanoate levels | Arachidonate (20:4n6) to caffeine ratio | Octadecenedioylcarnitine (C18:1-DC) levels | |  |  |
| 5alpha-androstan-3alpha,17beta-diol monosulfate (1) levels | Glycolithocholate sulfate levels | Glycoursodeoxycholic acid sulfate (1) levels | |  |  |
| Androsterone sulfate levels | Taurodeoxycholic acid 3-sulfate levels | Biliverdin levels |  |  |  |
| Imidazole lactate levels | Plasma free asparagine levels | Hexadecenedioate (C16:1-DC) levels |  |  |  |
| N-acetylkynurenine (2) levels | Phenylacetylglutamate levels | Hexadecanedioate (C16-DC) levels |  |  |  |
| Bilirubin (Z,Z) to etiocholanolone glucuronide ratio | Cis-3,4-methyleneheptanoylglycine levels | Aspartate to mannose ratio |  |  |  |
| Erythritol levels in elite athletes | Carnitine C5:1 levels | 2-hydroxy-4-(methylthio)butanoic acid levels | |  |  |
| Gamma-glutamylcitrulline levels | Sphingomyelin (d18:0/18:0, d19:0/17:0) levels | Octadecenedioate (C18:1-DC) levels |  |  |  |
| Behenoylcarnitine (C22) levels | Inosine to EDTA ratio | N,N,N-trimethyl-5-aminovalerate levels | |  |  |
| Cis-4-decenoate (10:1n6) levels | Dihomo-linoleoylcarnitine (C20:2) levels | Octadecadienedioate (C18:2-DC) levels |  |  |  |
| N-acetylleucine levels | Glycosyl-N-stearoyl-sphingosine (d18:1/18:0) levels | Glycocholenate sulfate levels |  |  |  |
| 5alpha-pregnan-3beta,20beta-diol monosulfate (1) levels | 1-(1-enyl-palmitoyl)-2-oleoyl-gpc (p-16:0/18:1) levels | Glycochenodeoxycholate glucuronide (1) levels | |  |  |
| Methyl glucopyranoside (alpha + beta) levels | Taurochenodeoxycholic acid 3-sulfate levels | 1-(1-enyl-palmitoyl)-2-palmitoyl-GPC (P-16:0/16:0) levels | |  |  |
| 5alpha-androstan-3beta,17beta-diol disulfate levels | Pregnenolone sulfate levels | Mannose to N-acetylglucosamine to N-acetylgalactosamine ratio | |  |  |
| Stearoyl sphingomyelin (d18:1/18:0) levels | Histidine to trans-urocanate ratio | Deoxycarnitine levels |  |  |  |
| 21-hydroxypregnenolone disulfate levels | N-acetylcarnosine levels | 1-ribosyl-imidazoleacetate levels |  |  |  |
| Adipoylcarnitine (C6-DC) levels | Cholesterol to oleoyl-linoleoyl-glycerol (18:1 to 18:2) [2] ratio | Glycolithocholate to glycolithocholate sulfate ratio | |  |  |
| Adenosine 5'-diphosphate (ADP) to 2'-deoxyuridine ratio | 16a-hydroxy DHEA 3-sulfate levels | Adenosine 5'-diphosphate (ADP) to N-palmitoyl-sphingosine (d18:1 to 16:0) ratio | | |  |
| Adenosine 5'-monophosphate (AMP) to acetoacetate ratio | Taurocholenate sulfate levels | Phosphate to oleoyl-linoleoyl-glycerol (18:1 to 18:2) [2] ratio | |  |  |
| Adenosine 5'-diphosphate (ADP) to sulfate ratio | 11beta-hydroxyandrosterone glucuronide levels | Androstenediol (3beta,17beta) monosulfate (2) levels | |  |  |
| Nisinate (24:6n3) levels | 1-linoleoyl-2-arachidonoyl-GPC (18:2/20:4n6) levels | Threonine to alpha-ketobutyrate ratio |  |  |  |
| N-palmitoylglycine levels | 1-methylxanthine levels | Suberate (C8-DC) levels |  |  |  |
| Retinol (Vitamin A) to linoleoyl-arachidonoyl-glycerol (18:2 to 20:4) [1] ratio | 3beta-hydroxy-5-cholestenoate levels | 2-hydroxysebacate levels |  |  |  |
| S-methylcysteine levels | Aspartate levels | 2-aminoheptanoate levels |  |  |  |
| Adenosine 5'-monophosphate (AMP) to glycine ratio | 1-arachidonoyl-GPE (20:4n6) levels | Tartronate (hydroxymalonate) levels |  |  |  |
| Proline to glutamate ratio | Spermidine to ergothioneine ratio | Sphingomyelin (d18:2/24:2) levels |  |  |  |
| Tetradecadienedioate (C14:2-DC) levels | Bilirubin degradation product, C16H18N2O5 (1) levels | N-acetylaspartate (naa) levels |  |  |  |
| Margaroylcarnitine (C17) levels | 5-acetylamino-6-formylamino-3-methyluracil levels | Acetoacetate levels |  |  |  |
| 5alpha-pregnan-3beta,20alpha-diol monosulfate (2) levels | Bilirubin degradation product, C16H18N2O5 (3) levels | S-methylmethionine levels |  |  |  |
| Theophylline levels | Bilirubin degradation product, C16H18N2O5 (4) levels | 4-methoxyphenol sulfate levels |  |  |  |
| Creatine to carnitine ratio | Oleoyl-linoleoyl-glycerol (18:1 to 18:2) [2] to linoleoyl-arachidonoyl-glycerol (18:2 to 20:4) [1] ratio | Cortisone to cortisol ratio |  |  |  |
| Stearoylcarnitine levels | Succinoyltaurine levels | Pantothenate levels |  |  |  |
| Pentadecanoate (15:0) levels | Bilirubin degradation product, C16H18N2O5 (2) levels | Salicylate to oxalate (ethanedioate) ratio | |  |  |
| N-acetyl-2-aminoadipate levels | N-acetylputrescine to (N(1) + N(8))-acetylspermidine ratio | P-cresol glucuronide levels |  |  |  |
| Stearoylcholine levels | Bilirubin degradation product, C17H20N2O5 (1) levels | Gamma-glutamyl-2-aminobutyrate levels | |  |  |
| Pantoate levels | N-acetylputrescine levels | Quinolinate levels |  |  |  |
| Vanillactate levels | N-palmitoyl-sphingosine (d18:1 to 16:0) to N-stearoyl-sphingosine (d18:1 to 18:0) ratio | 8-methoxykynurenate levels |  |  |  |
| 10-heptadecenoate (17:1n7) levels | Retinol (Vitamin A) to linoleoyl-arachidonoyl-glycerol (18:2 to 20:4) [2] ratio | Spermidine to histidine ratio |  |  |  |
| Plasma free proline levels | Bilirubin degradation product, C17H20N2O5 (2) levels | 1-palmitoyl-GPI (16:0) levels |  |  |  |
| Oleoylcholine levels | 5-methyluridine (ribothymidine) levels | 3-methoxytyrosine levels |  |  |  |
| Eicosenoate (20:1) levels | 1-stearoyl-2-oleoyl-GPI (18:0/18:1) levels | Ferulic acid 4-sulfate levels |  |  |  |
| Spermidine to choline ratio | Linoleoyl ethanolamide levels | Phosphate to N-palmitoyl-sphingosine (d18:1 to 16:0) ratio | |  |  |
| Mannose to fructose ratio | Alpha-hydroxyisovalerate levels | Hydroquinone sulfate levels |  |  |  |
|  | N6-acetyllysine levels | Hexanoylglutamine levels |  |  |  |
|  | 5-acetylamino-6-amino-3-methyluracil levels |  |  |  |  |
|  | Cholesterol to linoleoyl-arachidonoyl-glycerol (18:2 to 20:4) [1] ratio | |  |  |  |
|  | Benzoate to linoleoyl-arachidonoyl-glycerol (18:2 to 20:4) [2] ratio | |  |  |  |
|  | 1-(1-enyl-palmitoyl)-2-linoleoyl-GPE (p-16:0/18:2) levels | |  |  |  |
|  | Trans-urocanate levels |  |  |  |  |
|  | Adenosine 5'-monophosphate (AMP) to aspartate ratio | |  |  |  |
|  | 2-methylserine levels |  |  |  |  |
|  | 1-palmitoleoyl-2-linolenoyl-GPC (16:1/18:3) levels | |  |  |  |
|  | Indolelactate levels |  |  |  |  |
|  | Oleoyl-linoleoyl-glycerol (18:1/18:2) [2] levels |  |  |  |  |
|  | Benzoate to linoleoyl-arachidonoyl-glycerol (18:2 to 20:4) [1] ratio | |  |  |  |
|  | Glutamate to cysteine ratio |  |  |  |  |
|  | Adenosine 5'-monophosphate (AMP) to tryptophan ratio | |  |  |  |
|  | Palmitoleoylcarnitine (C16:1) levels |  |  |  |  |
|  | Glucose-to-mannose ratio |  |  |  |  |
|  | Ascorbic acid 2-sulfate levels |  |  |  |  |
|  | Alpha-ketoglutarate to aspartate ratio |  |  |  |  |
|  | 1-palmitoleoyl-GPC (16:1) levels |  |  |  |  |
|  | 2-hydroxy-3-methylvalerate levels |  |  |  |  |
|  | 1-palmitoyl-2-oleoyl-GPI (16:0/18:1) levels |  |  |  |  |
|  | Lignoceroylcarnitine (C24) levels |  |  |  |  |
|  | Glycodeoxycholate levels |  |  |  |  |
|  | Adenosine 5'-monophosphate (AMP) to glutamine ratio | |  |  |  |
|  | Myristoleate (14:1n5) levels |  |  |  |  |
|  | Spermidine to pyruvate ratio |  |  |  |  |
|  | 1-(1-enyl-stearoyl)-2-oleoyl-GPE (p-18:0/18:1) levels | |  |  |  |
|  | Taurochenodeoxycholate levels |  |  |  |  |
|  | Catechol glucuronide levels |  |  |  |  |
|  | Aconitate [cis or trans] levels |  |  |  |  |
|  | Uridine levels |  |  |  |  |
|  | (16 or 17)-methylstearate (a19:0 or i19:0) levels |  |  |  |  |
|  | 3-methyl-2-oxobutyrate levels |  |  |  |  |
|  | 2-hydroxydecanoate levels |  |  |  |  |
|  | 2,3-dihydroxy-5-methylthio-4-pentenoate (dmtpa) levels | |  |  |  |
|  | N-acetylvaline levels |  |  |  |  |
|  | Phosphate to phosphoethanolamine ratio |  |  |  |  |
|  | Phenylacetylglutamine levels |  |  |  |  |
|  | Proline to trans-4-hydroxyproline ratio |  |  |  |  |
|  | 2,2'-Methylenebis(6-tert-butyl-p-cresol) levels |  |  |  |  |
|  | 2-hydroxybutyrate/2-hydroxyisobutyrate levels |  |  |  |  |
