# Supplementary material for: Causal inference of metabolites in autoimmune liver diseases: A Mendelian randomization analysis
Source: Medicine (Baltimore). 2026 May 8;105(19):e48221. doi: 10.1097/MD.0000000000048221 (PMC13166632; doi:10.1097/MD.0000000000048221)
Supplement: Supplementary file 2 [file medi-105-e48221-s004.docx]

**Table S2** Mendelian randomization statistical pleiotropy analyses for the associations between metabolites and AILD risk.

| exposure | outcome | Egger intercept | se | Pvalue |
| --- | --- | --- | --- | --- |
| Plasma free proline levels | Autoimmune hepatitis | -0.039 | 0.033 | 0.246 |
| Spermidine to N-acetylputrescine ratio | Primary biliary cholangitis | 0.025 | 0.018 | 0.207 |
| Hexadecanedioate (C16-DC) levels | Primary sclerosing cholangitis | 0.034 | 0.017 | 0.062 |
